# The amplitudes and the structure of the charge density wave in YBCO

**DOI:** 10.1038/srep34551

**Published:** 2016-10-10

**Authors:** Y. A. Kharkov, O. P. Sushkov

**Affiliations:** 1 School of Physics, University of New South Wales, Sydney 2052, Australia

## Abstract

We find unknown *s*- and *d*-wave amplitudes of the recently discovered charge density wave (CDW) in underdoped cuprates. To do so we perform a combined analysis of experimental data for ortho-II YBa_2_Cu_3_O_*y*_. The analysis includes data on nuclear magnetic resonance, resonant inelastic X-ray scattering, and hard X-ray diffraction. The amplitude of doping modulation found in our analysis is 3.5 · 10^−3^ in a low magnetic field and *T* = 60 K, the amplitude is 6.5 · 10^−3^ in a magnetic field of 30T and *T* = 1.3 K. The values are in units of elementary charge per unit cell of a CuO_2_ plane. We show that the data rule out a checkerboard pattern, and we also show that the data might rule out mechanisms of the CDW which do not include phonons.

The recent discovery of the charge density wave (CDW) in YBCO and other cuprates gave a new twist to physics of high-T_*c*_ superconductivity. Existence of a new charge ordered phase has been reported in bulk sensitive nuclear magnetic resonance (NMR) measurements^1^,^2^,^3^, resonant inelastic X-ray scattering (RIXS)^4^,^5^,^6^, resonant X-ray scattering^7^ and hard X-ray diffraction (XRD)^8^. Additional non-direct evidence comes from measurements of ultrasound speed^9^ and Kerr rotation angle^10^.

While the microscopic mechanism of the CDW and its relation to superconductivity remains an enigma, there are several firmly established facts listed below, here we specifically refer to YBCO. (i) The CDW state arises in the underdoped regime within the doping range 0.08 ≤ *p* ≤ 0.13. (ii) The onset temperature of CDW at doping *p* ∼ 0.1 is *T*_*CDW*_ ≈ 150 K, which is between the pseudogap temperature T ^*^ and the superconducting temperature *T*_*c*_, *T*_*c*_ < *T*_*CDW*_ < T ^*^. (iii) The CDW “competes” with superconductivity, the CDW amplitude is suppressed at *T* < *T*_*c*_. Probably due to this reason the CDW amplitude at *T* < *T*_*c*_ is enhanced by a magnetic field that suppresses superconductivity. (iv) The CDW wave-vector is directed along the CuO link in the CuO_2_ plane. (v) The wave-vector *Q* ≈ 0.31 r.l.u. only very weakly depends on doping. (vi) The CDW is essentially two-dimensional in low magnetic fields, the correlation length in the *c*-direction is about one lattice spacing, while the in-plane correlation length is *ξ*_*a*,*b*_ ∼ 20 lattice spacings. (vii) In high magnetic fields (*B* > 15 T) and low temperatures (*T* < 50 K) the CDW exhibits three-dimensional correlations with the correlation length in the *c*-direction *ξ*_*c*_ ∼ 5 lattice spacings^11^,^12^. (viii) Ionic displacements in the CDW are about 10^−3^ *Å*^13^.

In spite of numerous experimental and theoretical works, there are two major unsolved problems in the phenomenology of the CDW. (i) The amplitude of the electron density modulation remains undetermined. (ii) The intracell spatial charge pattern is unclear, while there are indications from RIXS^14^ and from scanning tunneling microscopy^15^ that the pattern is a combination of *s*- and *d*-waves. The major goal of the present work is to resolve the open problems. We stress that in the present paper we perform combined analysis of experimental data to resolve the problems of the phenomenology, but we do not build a microscopic model of the CDW. While we rely on various data, the most important information in this respect comes from NMR. In particular we use the ortho-II YBCO data. Ortho-II YBCO (doping *p* ≈ 0.11) is the least disordered underdoped cuprate and hence it has the narrowest NMR lines. Development of the CDW with decreasing of temperature leads to the broadening of the quadrupole satellites in the NMR spectrum^1^,^2^,^3^. Below we refer the quadrupole satellites as NQR lines. Quite often the term “NQR” implies zero magnetic field measurements. We stress that it is not true in our case, NQR here means quadrupole satellites of NMR lines. The broadening is directly proportional to the CDW amplitude with the coefficients determined in ref. 16. So, one can find the CDW amplitude and this is the idea of the present analysis. Moreover, combining the data on copper and oxygen NMR we deduce the CDW intracell pattern within the CuO_2_ plane.

The second goal of the present work is “partially theoretical”. Based on the phonon softening data^17^ we are able to separate between two broad classes of possible mechanisms responsible for the formation of the CDW. (i) In the first class the CDW is driven purely by strongly correlated electrons which generate the charge wave. In this case phonons and the lattice are only spectators which follow electrons. (ii) In the second class, which we call “the Peierls/Kohn” scenario, both electrons and phonons are involved in the CDW development on equal footing. We argue that the phonon softening data^17^ potentially supports the second scenario.

The CDW implies modulation of electron charge density on copper and oxygen sites in the CuO_2_-planes. Our notations correspond to the orthorhombic YBCO, the axis c is orthogonal to the CuO_2_-plane, the in-plane axes *a* and *b* are directed perpendicular and parallel to the oxygen chains, respectively. Usually the CDW is described in terms of *s*-, *s*′-, and *d*-wave components with amplitudes *A*_*s*_, *A*_*s*′_, and *A*_*d*_, see e.g. refs 14,15 and 18. The *s*-wave component corresponds to the modulation of the population of Cu 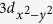 orbitals, and *s*′- and *d*-wave components correspond to the modulation of the populations of oxygen 2*p*_*σ*_ orbitals:


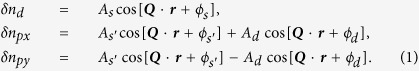


Here ***Q*** is the wave vector of the CDW, directed along *a* or *b* crystal axis [***Q*** = (*Q*, 0) or ***Q*** = (0, *Q*)] and *ϕ*_*s*_, *ϕ*_*s*′_, *ϕ*_*d*_ are the phases of *s*-, *s*′- and *d*-waves. The subscripts “*x*” and “*y*” in Eq. (1) indicate different oxygen sites within the CuO_2_-plane unit cell. The standard nomenclature of the oxygen sites in YBCO is O(2) and O(3). The O(2) 2p_*σ*_− orbital is parallel to the axis “*a*”, and the O(3) 2p_*σ*_- orbital is parallel to the axis “*b*”, see Fig. 1. For the CDW wave-vector ***Q*** directed along the *a*-axis, the “*x*”-site is O(2) and the “*y*”-site is O(3) as shown in Fig. 1. In the same figure we indicate excess charge corresponding to *s*, *s*′-, and *d*-waves. For ***Q*** orientated along the axis “*b*” the “*x*”-site is O(3), and the “*y*”-site is O(2).

According to the analysis^16^ the NQR frequency of a particular ^17^O nucleus is proportional to the local hole density *n*_*p*_ at this site, and of course it depends on the orientation of the magnetic field with respect to the oxygen p-orbital,


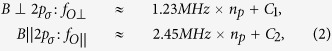


where *B* is the external magnetic field of NMR. Constants *C*_1_ and *C*_2_ are due to other ions in the lattice; generally they depend on the position of the oxygen ion in the lattice. Typical values of these constants are: *C*_1_ ∼ 0.2 MHz, *C*_2_ ∼ 0.5 MHz. According to the same analysis^16^ the ^63^Cu NQR frequency is proportional to the local hole density *n*_*d*_ at the Cu site and also *n*_*p*_ at the adjacent oxygen sites,


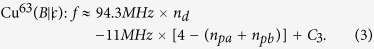


Here the “ion-related” constant C_3_ ∼ −6 MHz.

There are two mechanisms for the position dependent variation of the NQR frequency which are related to the CDW, (i) a variation of the local densities *n*_*d*_, *n*_*p*_, (ii) a variation of the ions’ positions. The position dependent frequency variation leads to the observed inhomogeneous broadening of the NQR line. Let us show that the mechanism (ii) is negligible. Only in-plane displacements of ions contribute to (ii) in the first order in the ion displacement. The magnitudes of the relative in-plane displacements of Cu and O ions are 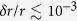 13, where *r* ≈ 2 *Å* is the Cu-O distance. Hence we can expect a lattice-related variation of e.g. oxygen *f*_⊥_ at the level *δf*_⊥_ ∼ *C*_1_*δr*/*r* ∼ 0.2 kHz. This is two orders of magnitude smaller than the CDW related broadening ∼10 kHz observed experimentally. For copper nuclei the expected ion-related broadening comes mainly from the 11 × 4 MHz term in (3), *δf* ∼ *δr*/*r* × 44 MHz ∼ 0.04 MHz. Again, this is much smaller than the observed broadening ∼1 MHz. These estimates demonstrate that one can neglect the contribution of the lattice distortion in the NQR broadening. Therefore, below we consider only the broadening mechanism (i) related to variation of hole densities.

Any compound has an intrinsic quenched disorder. The disorder is responsible for the NQR line widths at *T* > *T*_*CDW*_. The experimental NQR lines in a “weak magnetic field”, *B* = 12–15T, are practically symmetric, the analysis of the NQR lines and the corresponding values of full widths at half maximum (FWHM) are presented in refs 2 and 3. However, the experimental NQR lines in a “strong magnetic field”^1^, *B* ≈ 30T, are somewhat asymmetric due to various reasons. The asymmetry brings a small additional uncertainty in the analysis. The “strong field” data is less detailed than the “weak field” data and therefore the additional uncertainty is completely negligible, the “strong field” NQR widths are given in ref. 1. Hereafter we assume simple Gaussian lines, 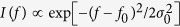, where *f*_0_ is the center of the NQR line, *σ*_0_ corresponds to the intrinsic disorder-related width. At *T* < *T*_*CDW*_ the line shape is changed to


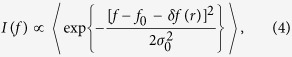


where *δf*(***r***),


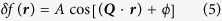


follows from Eqs (1, 2, 3). In particular, in MHz


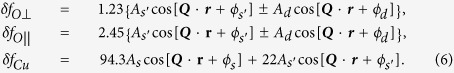


The averaging in Eq. (4), 〈....〉, is performed over the position *r* of a given ion (Cu or O) in the CuO plane. A simulation of *I*(*f*) in Eq. (4) with *δf* from (5) is straightforward, the results for several values of the ratio *A*/*σ*_0_ are presented in Fig. 2a. The CDW leads to the NQR line broadening and at larger amplitudes results in a distinctive double peak structure. For a comparison in the Panel b of Fig. 2 we present the lineshapes obtained with Eq. (4) for the checkerboard density modulation,


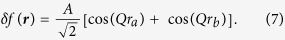


Obviously, the lineshapes in panels a and b of Fig. 2 are very different. The checkerboard pattern does not result in the double peak structure even at very large amplitudes. NQR data^1^,^2^ clearly indicate the double peak structure. This is a fingerprint of the stripe-like CDW. Comparison of the experimental NQR lineshapes with Panels b of Fig. 2 rules out the checkerboard scenario at large magnetic field, see also^19^,^20^,^21^.

The qualitative difference between the lineshapes corresponding to the stripe and the checkerboard patterns is a “density of states” effect. Indeed, the NQR intensity (4) can be written in terms of the “density of states”


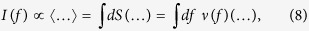


where (…) denotes the Gaussian exponent in (4), *dS* is the element of area in the CuO_2_ plane. The “density of states” *ν*(*f*) = ∫ *dS* *δ*(*f* − *f*_0_ − *δ* *f*(***r***)) in the case of the stripe-like CDW (5) has two singularities, see Fig. 3, at points *f* − *f*_0_ = ±*A*. The singularities result in two peaks in the NQR spectrum. On the other hand, in the case of the checkerboard CDW (7) the “density of states” *ν*(*f*) has a single maximum at *f* = *f*_0_, see Fig. 3, leading to a single-peak NQR lineshape. After averaging over the (*Q*, 0) and (0, *Q*) stripe domains the double-peak NQR lineshape is intact. Of course this is true only because the size of the domains (*ξ*_*a*,*b*_ ∼ 20 − 60 lattice spacings) is much larger then the period of the CDW (2*π*/*Q* ≈ 3.2 lattice spacings).

Numerical integration (averaging) in (4) shows that the full NQR line width at half maximum can be approximated as


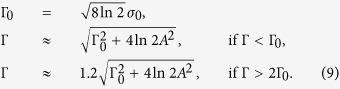


Note that this is the FWHM even for the non-Gaussian line shape like that in Fig. 2a. All the data we use below are in the regime Γ ≥ 2Γ_0_.

The typical dependence of the experimental NQR line width^3^ on temperature is sketched in Fig. 4. Hereafter we denote by Γ_0_ the value of the width at the temperature, where the width starts to increase with lowering of temperature, and by Γ_1_ the value of the width at the lowest available temperature, as indicated in Fig. 4. The increase of the width at low temperatures is due to the CDW development. Comparing the data with Eq. (9) we can find the CDW amplitudes. There are two distinct Cu positions in ortho-II YBCO, Cu(2E) and Cu(2F), that reside under the empty (E) and full(F) oxygen chains, respectively. There are also three distinct in-plane oxygen positions, O(2), O(3F), and O(3E). The O(2) 2*p*_*σ*_ orbital is oriented along the *a*-axis, and the O(3F), O(3E) 2*p*_*σ*_ orbitals are oriented along the *b*-axis (see Fig. 1). O(3F) resides under the full chain and O(3E) resides under the empty chain. The NQR broadening data for Cu(2E) and Cu(2F) are almost identical, the same is true for O(3F) and O(3E). Therefore, in our analysis we do not distinguish “E” and “F” and refer to them as Cu(2) and O(3) respectively.

It is worth noting that the NQR lines have been measured in NMR experiments. Therefore, the actual line broadening is a combined effect of the NQR broadening and the NMR broadening. The NMR broadening comes from the magnetic Knight shift which is proportional to the charge density modulation. The Knight shift broadening is itself an interesting effect which can bring additional information about CDW. However, our present analysis is aimed at NQR. The structure of NMR satellites enables the subtraction of the Knight shift effect from the data. The subtraction results in the “rectified” NQR line widths, which we use in our analysis. The rectified NQR line widths obtained in refs 3 and 22 for different ions and for different orientations of magnetic field are listed in the second and third columns of Table 1. In this case the magnetic field is *B* = 12 − 15T, Γ_0_ corresponds to 150 K and Γ_1_ corresponds to 60 K. Figures in brackets represent crude estimates of error bars.

The CDW-induced broadening at oxygen sites comes from contributions of the *s*′- and *d*-waves, see Eq. (6). RIXS and XRD data^4^,^5^,^6^,^8^ suggest that the CDW state consists of equally probable domains with the one-dimensional CDW along (*Q*, 0) and (0, *Q*) directions. This means, that even if the phases of *s*′ and *d*-wave are locked in a domain, say *ϕ*_*s*′_ = *ϕ*_*d*_, the *s*′-*d* interference disappears from the oxygen broadening after averaging over orientations of the domains. Hence, comparing Eqs (5) and (6) we conclude that for the oxygen sites 
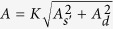
 with the coefficient *K* = 1.23 MHz or *K* = 2.45 MHz dependent on the orientation of the magnetic field. Using the experimental widths presented in Table 1 together with Eq. (9) one finds values of 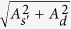 for each particular oxygen ion and orientation of the magnetic field. The values with indicative error bars are listed in the last column of Table 1. The average over the six different cases presented in the Table is


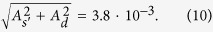


Note, that the indicative error bars in Table 1 are not statistical, therefore in Eq. (10) we present the simple average value.

The CDW-induced broadening at Cu sites comes from contributions of *s*- and *s*′-waves, see Eq. (6). Below we assume that the phases are locked, *ϕ*_*s*_ = *ϕ*_*s*′_. Hence comparing Eqs (5) and (6) we conclude that for Cu sites *A* = 94.3|*A*_*s*_ + 0.23*A*_*s*′_| MHz.

Using the experimental widths presented in the top line of Table 1 together with Eq. (9) we find


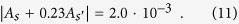


It is very natural to assume that the amplitudes *A*_*s*_ and *A*_*s*′_ are related as components of Zhang-Rice singlet, *A*_*s*_ ≈ 2*A*_*s*′_, see ref. 16. Hence, using (10), (11) we come to the following CDW amplitudes at *T* = 60 K and *B* ≈ 12 − 15T,


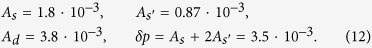


The values of *A*_*s*,*s*′,*d*_ are in units of the number of holes per atomic site, and *δp* is the doping modulation amplitude in units of the number of holes per unit cell of a CuO_2_ plane. Values of the amplitudes have not been reported previously, but the ratios have been deduced from the polarization-resolved resonant X-ray scattering^14^. Our ratio *A*_*s*′_/*A*_*d*_ ≈ 0.23 is reasonably close to the value *A*_*s*′_/*A*_*d*_ ≈ 0.27 obtained in ref. 14, however the ratio *A*_*s*_/*A*_*d*_ ≈ 0.47 is significantly larger than the value reported in ref. 14. Superficially our ratio *A*_*s*′_/*A*_*d*_ ≈ 0.23 is reasonably close to the value *A*_*s*′_/*A*_*d*_ ≈ 0.27 obtained in ref. 14, on the other hand the ratio *A*_*s*_/*A*_*d*_ ≈ 0.47 is significantly larger than the value reported in ref. 14. However, one has to be careful making a direct comparison of our results with ref. 14. The analysis^14^ assumes either *s* + *d* or *s*′ + *d* models, while we keep the three components (*s* + *s*′ + *d* model). For example, it is easy to check that the *s*′ + *d* model (*s* = 0) is inconsistent with the NQR data, so the agreement in the value *A*_*s*′_/*A*_*d*_ ≈ 0.27 is purely accidental. On the other hand, in principle we can fit the NQR data by the *s* + *d* model (*s*′ = 0), this results in *A*_*s*_/*A*_*d*_ ≈ 0.53 that is inconsistent with^14^.

The ratios of the CDW amplitudes *A*_*s*_/*A*_*d*_ ∼ 0.2, *A*_*s*′_/*A*_*d*_ ∼ 0.1 have been reported in STM measurements with BSCCO and NaCCOC^18^. Comparing these ratios (although measured in different cuprates) with our results we see that^18^ indicates dominance of the *d*-wave component, whereas in our analysis the *s*-wave amplitude is about a half of the *d*-wave amplitude. We do not have an explanation for the discrepancy between our results and REXS/STM measurements^14^,^18^, moreover REXS and STM are inconsistent with each other. The advantage of our analysis is that it is very simple and straightforward and, of course, NQR is a bulk probe.

Unfortunately, NQR data for magnetic field *B* ≈ 30T are not that detailed as that for *B* ≈ 12 − 15T. Nevertheless, based on the Cu NQR/NMR line broadening measured in ref. 1 and rectifying the Cu NQR line width (subtracting the Knight shift), we conclude that Γ_0_ = Γ_*T*=75*K*_ = 0.6 MHz and Γ_1_ = Γ_*T*=1.3*K*_ = 1.0 MHz. Hence, using the same procedure as that for the low magnetic field, we find |*A*_*s*_ + 0.23*A*_*s*′_| = 3.7 · 10^−3^. Again, assuming the Zhang-Rice singlet ratio, *A*_*s*_ ≈ 2*A*_*s*′_, we find the *s*-wave CDW amplitudes for *B* ≈ 30T and *T* = 1.3 K:


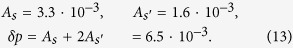


The doping modulation amplitude *δp* is about two times smaller than the estimate presented in ref. 1. Unfortunately, data^2^ are not sufficient for unambiguous subtraction of the Knight shift broadening from oxygen lines, so the determination of *A*_*d*_ is less accurate. However, roughly at *B* ≈ 30T and *T* = 1.3 K the value is *A*_*d*_ ∼ 6 · 10^−3^.

To complete our phenomenological analysis we comment on two broad classes of possible mechanisms of the CDW instability. (i) In the first class the CDW instability is driven purely by strongly correlated electrons which generate the charge wave. It can be due to electron-electron interaction mediated by spin fluctuations and/or due to the Coulomb interaction, see e.g. refs 23 and 24. We call this class of CDW formation mechanisms the “electronic scenario”. In this scenario phonons/lattice are not crucial for the CDW instability, they are only spectators that follow electrons. (ii) In the second class which we call the “Peierls/Kohn scenario” and which is known in some other compounds^25^,^26^,^27^, both electrons and phonons are involved in the CDW development on equal footing. We argue that the phonon softening data might support the second class.

A very significant softening of transverse acoustic and transverse optical modes in YBCO has been observed in ref. 17. The softening data are reproduced in Fig. 5a. The anomaly is very narrow in momentum space, *δq* ≈ 0.04 r.l.u., and it is only two times broader than the width of the elastic CDW peak, *δq* = 1/*ξ*_*a*,*b*_ ≈ 0.02 r.l.u. measured in RIXS and XRD^4^,^6^,^8^.

Our observation is very simple, in the case of the “electronic” scenario, the electronic CDW creates a weak periodic potential 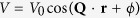 for phonons. Diffraction of phonons from the potential must lead to the usual band-structure discontinuity of the phonon dispersion *ω*_*q*_ at *q* = *Q* as it is shown by blue lines in Panel b of Fig. 5. In the presence of the finite correlation length of the CDW the discontinuity is healed as it is shown by the red solid line at the same panel, and combined blue-red solid line shows the expected phonon dispersion. In Supplementary material we present a calculation which supports this picture, but generally the picture is very intuitive. Obviously, this physical picture for the phonon dispersion is qualitatively different from the experimental data in Panel a of Fig. 5. On the other hand the “Peierls/Kohn scenario”, where both electrons and phonons are equally involved in the CDW development, leads to phonon dispersions like that in Fig. 5a. This has been observed in several compounds, see e.g. refs 25,27. Even though the phenomenological observation does not explain the mechanism of the CDW in underdoped cuprates, the observation poses a significant challenge to theoretical models based on “the electronic scenario” of the CDW formation. Furthermore, the phonon softening is generally expected in the “Kohn/Peierls scenario”, which is likely to be the case in YBCO. At the same time our arguments in favour of the “Kohn/Peierls scenario” are not quite conclusive. Indeed, it seems that the “Kohn/Peierls scenario” does not provide an explanation of the strong broadening of TA and TO phonon modes at *T* < *T*_*CDW*_^17^, as well as it does not explain why the phonon softening appears only in superconducting state *T* < *T*_*c*_. So the last point of our work is less solid than the main results concerning the amplitudes of the CDW. Nevertheless, we believe that the presented discussion of the “Kohn/Peierls scenario” versus “electronic scenario” is useful for future work on the microscopic mechanism of the CDW.

In conclusion, our analysis of available experimental data has resolved open problems in the phenomenology of the charge density wave (CDW) in underdoped cuprates. We have determined the amplitudes of *s*-, *s*′-, and *d*-wave components of the density wave. The amplitudes at low magnetic field and temperature *T* = 60 K are given in Eq. (12), and the amplitudes for magnetic field *B* = 30T and temperature *T* = 1.3 K are given in Eq. (13). We show that the data rule out a checkerboard pattern, and we also argue that the data might rule out mechanisms of the CDW which do not include phonons.

## Additional Information

**How to cite this article**: Kharkov, Y. A. and Sushkov, O. P. The amplitudes and the structure of the charge density wave in YBCO. *Sci. Rep.* **6**, 34551; doi: 10.1038/srep34551 (2016).

## Supplementary Material

Supplementary Information

## Figures and Tables

**Figure 1:**
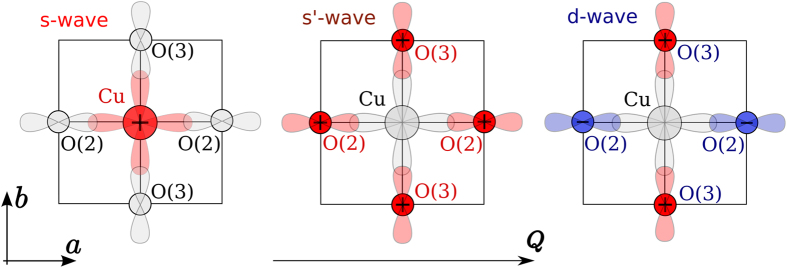
Intra-unit cell patterns of the CDW directed along the a-axis, *Q* = (*Q*, 0): *s*-wave, *s*′-wave, and *d*-wave. Positive and negative excess charge variations are shown in red and blue respectively.

**Figure 2:**
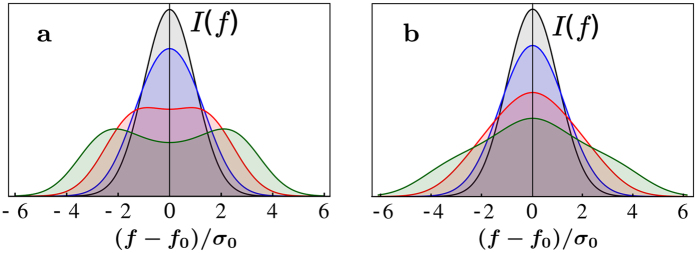
(**a**) The NQR lineshapes for the stripe-like CDW (5). **(b)** The NQR lineshapes for the checkerboard CDW (7). Both **(a)** and **(b)** show the lines for four different values of the CDW amplitude *A* with respect to the intrinsic broadening, *A*/*σ*_0_ = 0, 1, 2, 3.

**Figure 3:**
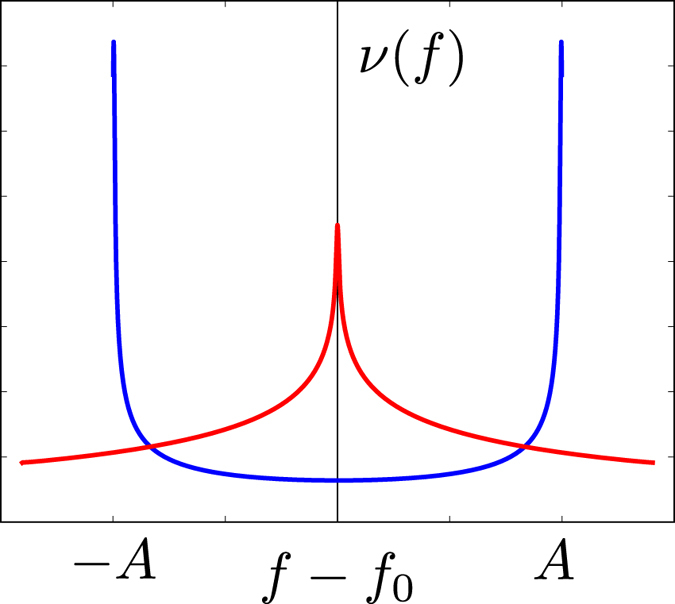
“Density of states” *ν*(*f*) for the stripe CDW (blue line) and for the checkerboard CDW (red line). The two singularities at *f* − *f*_0_ = ±*A* in the case of the stripe-like CDW result in the double peak structure of the NQR lines.

**Figure 4:**
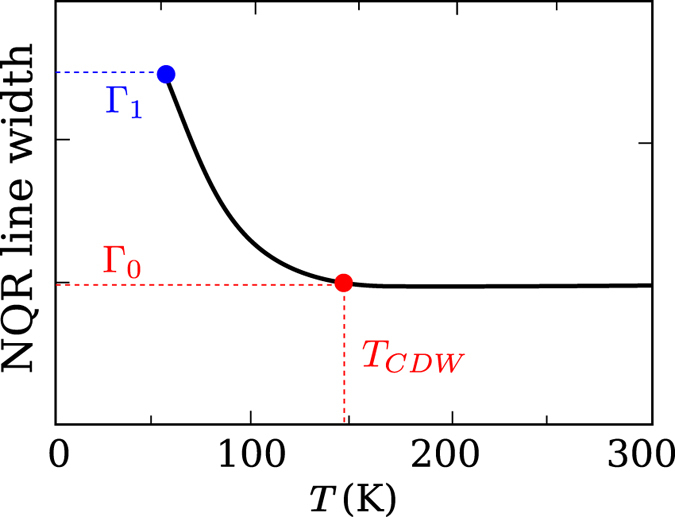
A schematic sketch of the temperature dependence of the NQR line width^3^. Γ_0_ is the width at the temperature *T*_*CDW*_, where the broadening starts to increase with temperature lowering, and Γ_1_ is the width at the lowest available temperature.

**Figure 5:**
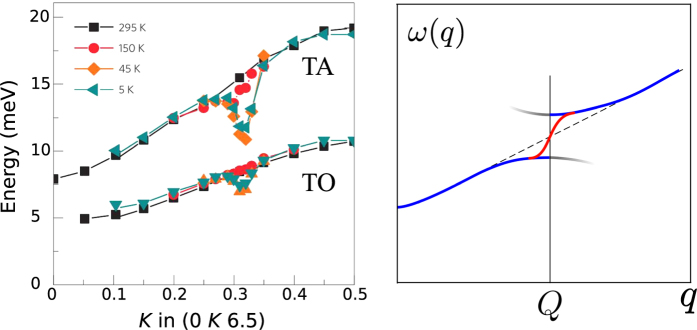
(**a**) Phonon anomaly (dip) in the dispersion of transverse acoustic (TA) and transverse optical (TO) modes at *q* = (0, *Q*, 6.5) in YBa_2_Cu_3_O_6.6_^17^. **(b)** The expected phonon dispersion in the “electronic scenario”. Blue solid lines show the dispersion in a perfect long-range CDW, grey lines represent the shadow bands. For a finite CDW correlation length the shadow bands practically disappear and solid blue lines become connected by the red solid line.

**Table 1:** NQR data for ortho-II YBCO in magnetic field *B* = 12 − 15T.

	Γ_0_(*kHz*)	Γ_1_(*kHz*)	|*A*_*s*_ + 0.23*A*_*s*′_|
Cu(2), B||c	230(30)	460(80)	2.0(0.5)
			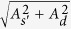
O(2), 2*p*_*σ*_||*a*, B||a	6.0(0.5)	16.0(1.3)	2.9(0.3)
O(2), 2*p*_*σ*_||*a*, B||b	6.0(0.6)	15.0(0.8)	5.4(0.4)
O(2), 2*p*_*σ*_||*a*, B||c + 20^*o*^	5.0(2.0)	16.0(2.5)	6.0(1.1)
O(3), 2*p*_*σ*_||*b*, B||a	6.0(1.0)	11.0(1.8)	3.6(0.9)
O(3), 2*p*_*σ*_||*b*, B||b	5.0(2.0)	12.0(2.0)	2.1(0.5)
O(3), 2*p*_*σ*_||*b*, B||c + 20^*o*^	9.0(2.3)	18.0(2.3)	6.1(1.4)

The line widths, Γ_0_ = Γ_*T*=150*K*_ and Γ_1_ = Γ_*T*=60*K*_, are measured for different ions and for different orientations of the magnetic field^3^,^22^.

The last column displays the CDW amplitudes deduced from the particular line. Figures in brackets represent crude estimates of error bars.
