# Supplementary material for: The amplitudes and the structure of the charge density wave in YBCO
Source: Sci Rep. 2016 Oct 10;6:34551. doi: 10.1038/srep34551 (PMC5056359; doi:10.1038/srep34551)
Supplement: Supplementary Information [file srep34551-s1.pdf]

Supplementary material to “The amplitudes and the structure of the charge density wave in YBCO”

Y. A. Kharkov and O. P. Sushkov*
School of Physics, University of New South Wales, Sydney 2052, Australia

A. Ginzburg-Landau model of the CDW and phonon dispersion

Here we consider a simple phenomenological Ginzburg-Landau model of the CDW developed in purely electron sector. Phonons weakly interact with electrons and follow the developed CDW as “spectators”. This is the first scenario discussed in the main text. Our analysis below shows that the phonon softening data does not support this scenario.

Let us consider a quasi-one dimensional (stripe-like) CDW, we direct the x axis along the CDW wave-vector. The CDW can be represented as a collective bosonic mode (ψ) in the electronic system. The effective Ginzburg-Landau-like Lagrangian for the CDW mode ψ reads

$$\mathcal{L}_\psi = \frac{\dot{\psi}^2}{2} - \psi\frac{\hat{\Omega}^2}{2}\psi - \frac{\alpha}{4}\psi^4 \ , \tag{1}$$

where $\psi(r)$ is a variation of electron density, $\hat{\Omega}^2/2$ is operator of “stiffness” of the CDW mode, and $\alpha > 0$ is a self-action constant. In momentum representation Ω_q^2 is a simple function sketched in Fig. 1. Kinetic energy has

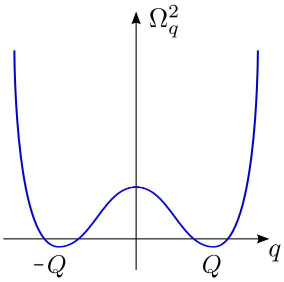

Figure 1: A sketch of the effective kinetic energy Ω_q^2 of the electronic CDW mode ψ.

minima at $q_x = \pm Q$, and we can represent it as

$$\Omega_q^2 = \Omega_Q^2 + \kappa(q_x^2 - Q^2)^2/4, \tag{2}$$

where κ is a some constant. Importantly the minimal value of $\Omega_{\pm Q}^2$ is negative, $\Omega_{\pm Q}^2 < 0$, providing formation of the incommensurate CDW with the wave vector Q. The density variation is real, hence

$$\psi(x) = 2|\psi_Q|\cos(Qx + \phi), \tag{3}$$

here ψ_Q is a Fourier component of $\psi(r)$. In a perfect system the phase ϕ is arbitrary and this must result in a Goldstone sliding mode. Of course a disorder pins the phase. A similar phenomenological approach was successfully applied to describe a CDW state in transition-metal dichalcogenides [1].

To find the CDW amplitude one has to minimize the energy

$$E_\psi = \int d^2r \left(\psi\frac{\hat{\Omega}^2}{2}\psi + \frac{\alpha}{4}\psi^4\right) . \tag{4}$$

---

*Electronic address: sushkov@unsw.edu.au

The saddle-point equation for static $\psi(r)$ reads

$$\hat{\Omega}^2\psi + \alpha\psi^3 = 0. \tag{5}$$

Performing Fourier transform in Eqs. (5) and leaving only the dominating Fourier components with $q = \pm Q$ we find

$$|\psi_Q|^2 = -\Omega_Q^2/3\alpha. \tag{6}$$

To find the CDW excitation spectrum we expand energy (4) up to second order in fluctuations on the top of the ground state (3),(6),

$$\delta^2 E_\psi = \int d^2r \left(\delta\psi\frac{\hat{\Omega}^2}{2}\delta\psi + \frac{3\alpha}{2}\psi^2(\delta\psi)^2\right). \tag{7}$$

The term $\propto \psi^2 = |\psi_Q|^2(2 + e^{2i(Qx+\phi)} + e^{-2i(Qx+\phi)})$ in (7) plays role of the effective potential with wave vector $2Q$ for the ψ-excitations. This results in a mixing between $\delta\psi_q$ and $\delta\psi_{q-2Q}$ (hereafter we assume that $q > 0$). Therefore, it is convenient to write the excitation energy as

$$\delta^2 E_\psi = \sum_q \Psi_q^\dagger \begin{pmatrix} \Omega_q^2 + 2|\Omega_Q^2| & |\Omega_Q^2|e^{2i\phi} \\ |\Omega_Q^2|e^{-2i\phi} & \Omega_{q-2Q}^2 + 2|\Omega_Q^2| \end{pmatrix} \Psi_q, \tag{8}$$

where

$$\Psi_q = \begin{pmatrix} \delta\psi_q \\ \delta\psi_{q-2Q} \end{pmatrix}.$$

Euler-Lagrange equation corresponding to (1), (8) results in the two normal modes: the sliding Goldstone mode and the gapped Higgs mode with the energies

$$\begin{aligned} \epsilon_{Gq} &= \sqrt{\kappa Q^2}|p_x|, \\ \epsilon_{Hq} &= \sqrt{2|\Omega_Q^2| + \kappa Q^2 p_x^2} \\ \boldsymbol{p} &= \boldsymbol{q} - \boldsymbol{Q}\ . \end{aligned} \tag{9}$$

The corresponding eigenmodes are

$$\begin{aligned} G_q &= 1/\sqrt{2}(e^{i\phi}\delta\psi_q - e^{-i\phi}\delta\psi_{q-2Q}), \\ H_q &= 1/\sqrt{2}(e^{i\phi}\delta\psi_q + e^{-i\phi}\delta\psi_{q-2Q}). \end{aligned} \tag{10}$$

Interestingly, due to the parabolic behaviour of Ω_q^2 near $q_x = \pm Q$, see Fig. 1, the weights of the states with wave vectors q and $q - 2Q$ in (10) are equal in a broad range of momenta around $q = \pm Q$.

The phonon is described by field φ, the phonon Lagrangian reads

$$\mathcal{L}_\varphi = \frac{\dot{\varphi}^2}{2} - \varphi\frac{\hat{\omega}^2}{2}\varphi, \tag{11}$$

where $\hat{\omega}^2/2$ is the elastic energy of lattice deformation. In momentum representation it is equivalent to the bare phonon dispersion ω_q. The CDW and phonons weakly interact, we describe the interaction by the Lagrangian

$$\mathcal{L}_{int} = -\lambda\psi\varphi\ , \tag{12}$$

where λ is coupling constant. Due to the coupling the CDW creates phonon condensate at $q_x = \pm Q$ (static lattice deformation) with amplitude $\varphi_Q = -\lambda/\omega_Q^2\,\psi_{-Q}$. Let us now consider phonon dispersion in the presence of the collective CDW mode. The interaction (12) in combination with Eqs (10) results in the following vertexes describing transition of phonon to the Higgs and Goldstone modes of the CDW.

$$\begin{aligned} \langle\delta\varphi_q|H_q\rangle &= \langle\delta\varphi_q|G_q\rangle = \frac{\lambda}{\sqrt{2}}e^{i\phi} \\ \langle\delta\varphi_{q-2Q}|H_q\rangle &= -\langle\delta\varphi_{q-2Q}|G_q\rangle = \frac{\lambda}{\sqrt{2}}e^{-i\phi}\ . \end{aligned}$$

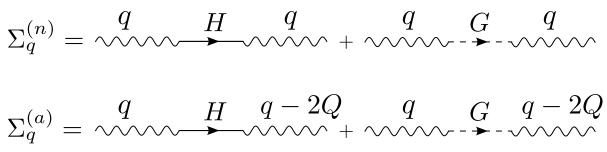

Figure 2: Normal and anomalous phonon self-energy

This leads to normal and anomalous phonon self-energy operators shown in Fig. 2. The corresponding analytical expressions are

$$\begin{aligned}
\Sigma_q^{(n)} &= \frac{\lambda^2}{2}\left(\frac{1}{\omega_q^2-\epsilon_{Hq}^2}+\frac{1}{\omega_q^2-\epsilon_{Gq}^2}\right),\\
\Sigma_q^{(a)} &= e^{2i\phi}\Delta_q^2,\\
\Delta_q^2 &= \frac{\lambda^2}{2}\left(\frac{1}{\omega_q^2-\epsilon_{Hq}^2}-\frac{1}{\omega_q^2-\epsilon_{Gq}^2}\right).
\end{aligned} \tag{13}$$

A selfenergy generally depends on q and ω. In Eqs.(13) we set $\omega = \omega_q$. So "renormalized" dispersion $\widetilde{\omega}_q$ of phonons is described by the eigenvalue problem

$$\widetilde{\omega}_q^2 \Phi_q = \begin{pmatrix} \omega_q^2 + \Sigma_q^{(n)} & e^{2i\phi}\Delta_q^2 \\ e^{-2i\phi}\Delta_q^2 & \omega_{q-2Q}^2 + \Sigma_{q-2Q}^{(n)} \end{pmatrix} \Phi_q \ , \tag{14}$$

where

$$\Phi_q = \begin{pmatrix} \delta\varphi_q \\ \delta\varphi_{q-2Q} \end{pmatrix}. \tag{15}$$

As it is intuitively clear even without a calculation Eq. (14) is equivalent to scattering of phonon from some effective periodic potential with wave vector $2Q$.

One can represent matrix elements in (14) as

$$\begin{aligned}
&\sqrt{\omega_q^2 + \Sigma_q^{(n)}} \approx w + cp_x,\\
&\sqrt{\omega_{q-2Q}^2 + \Sigma_{q-2Q}^{(n)}} \approx w - cp_x,\\
&\Delta_q \approx \Delta \ ,
\end{aligned} \tag{16}$$

when the detuning $\boldsymbol{p} = \boldsymbol{q} - \boldsymbol{Q}$ is small. The speed c follows from the overall slope of the phonon dispersion. For the TO mode $w \approx 15$ meV, $c \approx 30$ meV/r.l.u., see Fig. 5a in the main text. The phonon dispersion which follows from (14) and (16),

$$\widetilde{\omega}_q^2 \approx w^2 \pm \sqrt{4w^2c^2p_x^2 + \Delta^4} \ , \tag{17}$$

is shown in Fig.5b in the main text by the blue solid line. Shadow bands are indicated by fading grey lines. Expected intensity of the shadow bands is extremely small. Intensities of the bright (br) and shadow (sh) modes are

$$\begin{aligned}
I_{br} &\propto |\delta\varphi_q|^2 = \frac{1}{2}\left(1 + \frac{2wcp_x}{\sqrt{4w^2c^2p_x^2+\Delta^2}}\right),\\
I_{sh} &\propto |\delta\varphi_{q-2Q}|^2 = \frac{1}{2}\left(1 - \frac{2wcp_x}{\sqrt{4w^2c^2p_x^2+\Delta^2}}\right).
\end{aligned}$$

If the gap in the phonon spectrum ($gap \approx \Delta^2/w$) is 3 meV (TO mode), the intensity of the shadow mode practically diminishes at detuning $p_x = q_x - Q \geq 0.04$ r.l.u.

So far we disregarded temperature and intrinsic disorder. We remind the following experimental observations: (i) the CDW onset temperature is $T_{CDW} \sim 150$K, (ii) the CDW in low/zero magnetic fields is essentially two-dimensional,

the correlation length in the c-direction is about one lattice spacing while the in-plane correlation length is $\xi_{a,b} \sim 20$ lattice spacings. In agreement with Mermin-Wagner theorem the observation (ii) implies that onset of the CDW at $T = T_{CDW}$ is not a true phase transition, it is a two-dimensional freezing crossover. Hence at $T > T_{CDW}$ the phase ϕ fluctuates with time and as a result the off-diagonal matrix element in Eq. (14) is averaged to zero, $e^{2i\phi}\Delta_q^2 \to 0$. Hence the phonon dispersion at $T > T_{CDW}$ near $q_x = +Q$ is

$$\widetilde{\omega}_q = \sqrt{\omega_q^2 + \Sigma_q^{(n)}} \approx w + cp_x \ . \tag{18}$$

At $T < T_{CDW}$ the temporal fluctuations freeze, however due to the quenched disorder the phase $\phi(r)$ is a fluctuating function of coordinate r with the correlation length $\xi_{a,b}$. If $|p_x| > 1/\xi_{a,b} = 0.02$ r.l.u. the spatial fluctuations are not relevant and the phonon dispersion is given by Eq.(17). However, if the detuning is small, $|p_x| \ll 1/\xi_{a,b}$, one must average over spatial fluctuations of the phase ϕ effectively vanishing the off-diagonal matrix element, $e^{2i\phi}\Delta_q^2 \to 0$. This results in the red solid line connecting the blue solid lines in Fig. 5b in the main text.

All in all the phonon dispersion expected if the "electronic" scenario is realized is shown in Fig. 5b (main text). Obviously, the dispersion is inconsistent with the data. Therefore, we rule out the "electronic" scenario.

[1] McMillan, W. L., Landau theory of charge-density waves in transition-metal dichalcogenides, *Phys. Rev. B* **12**, 1187-1196 (1975).
